# Rare case of malignant transformation of recurrent respiratory papillomatosis associated with human papillomavirus type 6 infection and p53 overexpression

**DOI:** 10.1186/2193-1801-2-153

**Published:** 2013-04-10

**Authors:** Takeharu Kanazawa, Noriyoshi Fukushima, Shoichiro Imayoshi, Takafumi Nagatomo, Kazumi Kawada, Hiroshi Nishino, Kiyoshi Misawa, Keiichi Ichimura

**Affiliations:** 1 Department of Otolaryngology/Head and Neck Surgery, Jichi Medical University School of Medicine, 3311-1 Yakushiji, Shimotsuke, Tochigi, 329-0498 Japan; 2 Department of Pathology, Jichi Medical University School of Medicine, Shimotsuke, 329-0498 Japan; 3 Department of Otolaryngology/Head and Neck Surgery, Hamamatsu University School of Medicine, Hamamatsu, 431-3192 Japan

**Keywords:** Human papillomavirus, Malignant transformation, p53, Immunohistochemistry, Recurrent respiratory papillomatosis

## Abstract

Recurrent respiratory papillomatosis (RRP), a chronic upper respiratory condition characterized by diffuse multiple recurring papillomas, is thought to result from human papillomavirus (HPV) type 6 or 11 infection. Although RRP is an intractable disease, malignant transformation of RRP is rare. The underlying mechanism, however, has not been elucidated. We describe the clinical course of a patient who underwent more than 130 operations for RRP associated with HPV type 6 infection and subsequently suffered spontaneous malignant transformation to squamous cell carcinoma. Immunohistochemical analysis revealed that malignant transformation might result from a genomic defect, such as p53 inactivation, leading to stimulation of uncontrolled cell proliferation by HPV type 6 for an extended period, but not directly because of HPV itself. Our results could help in the development of novel therapeutic strategies for severe RRP, although further studies are required before clinical application of molecular targeted therapies.

## Introduction

Recurrent respiratory papillomatosis (RRP), usually a benign disease, is a chronic upper respiratory condition characterized by the occurrence of diffuse multiple recurring papillomas, which are caused by infection with human papillomavirus (HPV) type 6 and/or 11 (Go et al. 2003; Katsenos & Becker 2011). RRP has a bimodal age distribution with peaks of incidence at 5 and 20–30 years of age, corresponding to juvenile-onset and adult-onset RRP, respectively (Hall et al. 2011). In juvenile-onset cases, a number of different drugs have previously been used as adjuvant therapy, including antiviral agents, interferon, retinoids (Katsenos & Becker 2011; Gallagher & Derkay 2009) and cidofovir, but their efficacies are limited (Shehab et al. 2005; Kimberlin & Malis 2000). Surgical removal of respiratory tract papilloma is the primary treatment for RRP, but it is also used as palliative treatment for the prevention of severe dyspnea and stridor (Bergler & Gotte 2000; Preuss et al. 2007). Thus, many patients require multiple surgeries and each episode of surgery or surgical trauma can result in the reemergence or worsening of the papillomas (Preuss et al. 2007).

Both HPV types 6 and 11, which cause RRP, are considered to be low-risk oncogenic HPVs, and malignant transformation is a rare occurrence. Data from clinical cases has however indicated that HPV type 11 is associated with a greater risk of malignant transformation than HPV type 6 (Reidy et al. 2004; Gerein et al. 2005).

Here, we report a case of RRP that underwent spontaneous malignant transformation to squamous cell carcinoma (SCC) after more than 130 operations, and which was, unusually, associated with HPV type 6 infection. We also identified protein markers of proliferation and neoplastic change in order to help elucidate the mechanism of malignant transformation in this case.

## Case presentation

The patient in this case was a 39-year-old Japanese man with a history of RRP, who had presented with progressive hoarseness when he was 1 year old and was diagnosed with laryngeal papilloma by biopsy under general anesthesia 3 years later. Surgical excision and laser ablation were performed, but the papillomas immediately recurred and resulted again in severe dyspnea. When the patient was 6 years old, a tracheotomy was performed to relieve severe dyspnea. During this procedure, a large papilloma was discovered. RRP was diagnosed, and the patient was followed-up at our hospital. Initially, various treatments were tried including interferon-α, interferon-β, interferon-γ, acyclovir, fluorouracil, retinoids, and traditional Chinese medicines, but none of these treatments resulted in a significant clinical response.

The tumors originated in the larynx, gradually increased in size, and progressed to the tracheal bifurcation, although the pathological diagnosis was still one of mild dysplasia. Thus, treatment was continued in the form of repeated surgical reductions of the papilloma using an endoscopic microdebrider or potassium-titanyl-phosphate (KTP) laser to maintain the airway over the next 35 years. This involved more than 130 procedures. In 2011, granulation tissue around the tracheotomy aperture was found to have increased and a biopsy revealed indicated SCC. A larynx biopsy performed at the same time revealed additional moderate dysplasia but not malignancy. This patient received irradiation by accelerated hyperfractionation resulting in good local control, but he died from metastasis to the lung.

RRP is a potentially devastating disease that can be associated with significant morbidity, and result in mortality due to airway compromise even if not change to malignant. However, malignant transformation is not commonly seen, and thus, the underlying mechanism is still unclear. In an attempt to determine the mechanism of malignant transformation in this case, HPV typing and diachronic immunohistochemistry were performed. HPV typing by multiplex-PCR detected HPV type 6 infection but not evidence of “high-risk” type HPV infection.

**Figure 1:**
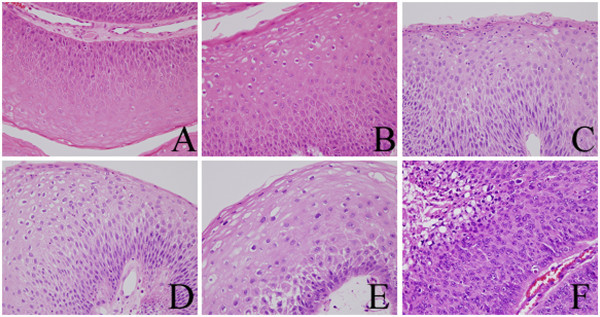
**Hematoxylin eosin stain.** The specimen obtained from the larynx in 1989 (**A**), 1994 (**B**), 2000 (**C**), 2005 (**D**), and 2011 (**E**), and from the tumor tissue around the tracheotomy aperture in 2011 (**F**).

**Figure 2:**
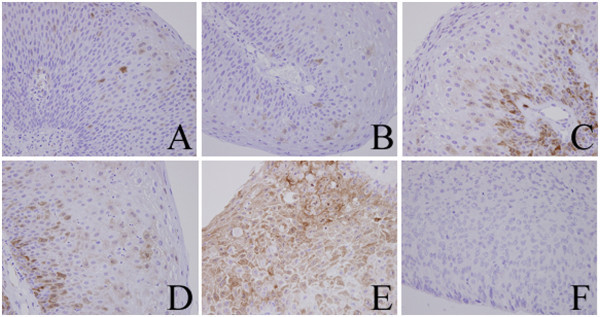
**Immunohistochemical analysis of p16.** The specimens obtained from the larynx in 1989 (**A**), 1994 (**B**), 2000 (**C**), 2005 (**D**),and 2011 (**E**), and from the tumor tissue around the tracheotomy aperture in 2011 (**F**).

**Figure 3:**
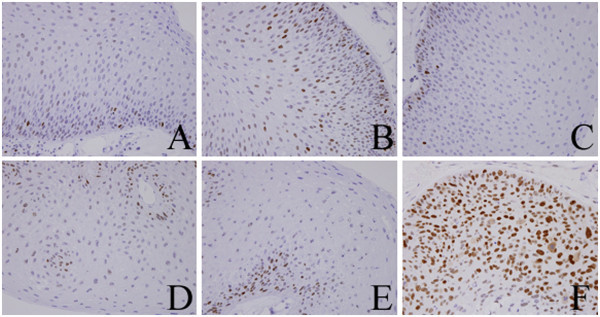
**Immunohistochemical analysis of p53.** The specimens obtained from the larynx in 1989 (**A**), 1994 (**B**), 2000 (**C**), 2005 (**D**),and 2011 (**E**), and from the tumor tissue around the tracheotomy aperture in 2011 (**F**).

We performed immunohistochemistry on specimens obtained from the larynx in 1989, 1994, 2000, 2005, and 2011, and on the tumor tissue obtained from around the tracheotomy aperture in 2011. All specimens from the larynx showed mild to moderate dysplasia (Figure 1A, B, C, D, and E), but the specimen from around the tracheotomy aperture showed SCC (Figure 1F) on hematoxylin and eosin (HE) staining. Diachronic HE staining revealed that there was no progress of dysplasia towards malignancy until a SCC was detected in the tissue around the tracheotomy aperture in 2011. The level of p16 staining diachronically increased in parts of the dysplastic tissue (Figure 2A, B, C, D, and E) but p16 staining was not positive in the SCC removed from the tumor tissue around the tracheotomy aperture (Figure 2F). In addition, the dysplastic tissue was negative for p53 staining (Figure 3A, B, C, D, and E), but the SCC showed strong positive staining for this marker (Figure 3F).

**Figure 4:**
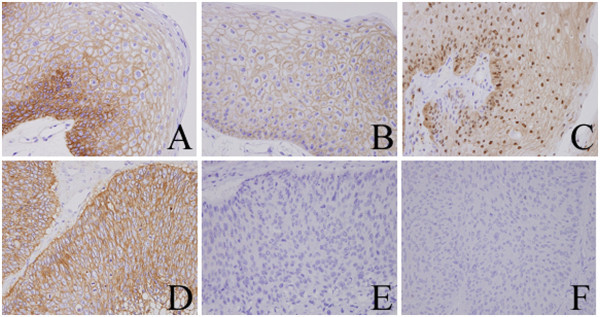
**Immunohistochemical staining for EGFR signaling.** EGFR (**A**), pEGFR (**B**), and pERK (**C**) in the dysplastic sample obtained in 2011, and for EGFR(**D**), pEGFR (**E**), and pERK (**F**) in the SCC sample.

Both the dysplastic tissue and the SCC stained strongly positive for epidermal growth factor receptor (EGFR, Figure 4A and D). In order to investigate possible differences in EGFR signaling between the dysplastic tissue and the SCC, we also used immunohistochemistry to detect the key proteins of the EGFR signaling pathway: phosphorylated EGFR (pEGFR), phosphorylated extracellular signal-regulated kinase (pERK), and phosphorylated AKT (pAKT). Staining was positive for pEGFR (Figure 4B) and pERK (Figure 4C) and negative for pAKT in the dysplastic tissue, whereas staining for pEGFR (Figure 4E), pERK (Figure 4F), and pAKT was negative in the SCC. Results of immunohistochemistry were summarized in Table 1.

**Table 1:** **Summary of immunohistochemistry**

	A	B	C	D	E	F
HE	dysplasia	dysplasia	dysplasia	dysplasia	dysplasia	SCC
p16	mild	mild	moderate	moderate	strong	negative
p53	mild	mild	mild	mild	mild	strong
EGFR	strong	strong	strong	strong	strong	strong
pEGFR					positive	negative
pERK					positive	negative

Specimens obtained from the larynx in 1989 (A), 1994 (B), 2000 (C), 2005 (D), and 2011 (E), and on the tumor tissue obtained from around the tracheotomy aperture in 2011 (F).

## Discussion

RRP is a rare condition characterized by recurrent growth of benign papillomas in the respiratory tract, and is now known to be caused exclusively by HPV, usually type 6 and/or 11 (Go et al. 2003; Katsenos & Becker 2011). The disease presents in 2 forms according to age at onset. Juvenile RRP, which occurs in patients under 5 years, is considered more aggressive than the adult type (Hall et al. 2011). Juvenile RRP is a severe and potentially fatal disease because it can result in airway obstruction on multiple recurrence (Kimberlin & Malis 2000).

Various treatments for RRP using antiviral therapies including interferon, retinoids, photodynamic therapy, zinc, anti-reflux medications, cyclooxygenase-2 inhibitors, therapeutic/preventive vaccines, and gene therapy have been trialled (Katsenos & Becker 2011; Gallagher & Derkay 2009). One therapy in particular, intralesional injection of the anti-viral agent cidofovir, has shown particular promise (Shehab et al. 2005; Kimberlin & Malis 2000). However, it is known that cidofovior has a malignant transformation risk and the link between cidofovir and malignancy is very currently topic, thus it is not covered by National Health Insurance in Japan and we could not use it. Although other various treatments were used in this case, few curative effects were observed. This case required more than 130 surgical debulking procedures using an endoscopic microdebrider or KTP laser to maintain the airway over a period of 35 years.

RRP is considered to be an intractable disease as it generally does not respond to therapeutic interventions, but malignant transformation is very rare, occurring in only 1–4% of patients with RRP (Jeong et al. 2009). A malignant transformation rate of 1.7% was reported in a series of 179 patients (Klozar et al. 1997), and malignant transformation or secondary airway carcinoma were observed in 4% of 194 patients in another large-scale study (Preuss et al. 2007).

The molecular mechanisms that underlie the malignant transformation of RRP is still unknown, but risk factors include smoking, prior irradiation, juvenile onset, and the type of infecting HPV (Katsenos & Becker 2011). HPV types 6 and 11 are the most frequent detectable genotypes in RRP specimens, but HPV type 11-positive patients are characterized by more aggressive RRP, a more frequent requirement for tracheotomy, malignant transformation, and mortality during follow-up (Gerein et al. 2005).

To date, two mechanisms for malignant transformation of RRP associated with HPV type 11 infection have been reported. Reidy et al. showed that HPV type 11 genes can integrate into the host genome, and the three oncogenic proteins, E5, E6, and E7 produced by the integrated HPV have been implicated as malignant transforming factors (Reidy et al. 2004). This process is the same as that known to underlie the malignant conversion of “high-risk” types of papillomavirus in cervical intraepithelial neoplasia. Products of the E6 and E7 genes degrade p53 and retinoblastoma protein, and the E5 gene product stimulates EGFR activation to induce cell proliferation (Kim et al. 2010). Other studies also demonstrated that p53 is overexpressed in the carcinoma component of RRP associated with HPV type 11 infection, suggesting the presence of another mechanism (Go et al. 2003; Rady et al. 1998; Stern et al. 2000). Previous reports have mainly examined the mechanism of malignant transformation in RRP resulting from HPV type 11 infection, and only a few concerned RRP associated with HPV type 6 infection.

The patient in the present case was diagnosed with juvenile RRP associated with infection by HPV type 6, with no history of smoking and previous irradiation, making this a very unusual case of malignant transformation. There are a number of possible explanations for the occurrence of malignant transformation in this relatively low-risk case, including re-infection by “high-risk” HPVs, an enhancement of the oncogenic potential of the existing HPV type 6 infection, the activation of oncogenic proteins in the papilloma cells because of increased cellular proliferation, or an inhibition of tumor suppressor genes.

In this study, we evaluated the expression of p16, known to be a surrogate marker for “high risk” HPV infection but not yet ill-defined for “low-risk” HPV infection. The strength of p16 staining was diachronically increased in the dysplastic tissue, but p16 staining was negative in the SCC. This suggests that the multiplicity of infection or the oncogenic potential of HPV was diachronically increased if p16 staining acts as a surrogate marker, but the malignant transformation might not have been a result of HPV infection. Furthermore, EGFR, a known oncogene in head and neck cancer that is activated by the product of the HPV E5 gene (Kim et al. 2010), was strongly expressed in the initial papilloma stage and at all subsequent stages through to SCC. However, only the dysplastic tissue was positive for pEGFR and pERK staining, indicating that the EGFR signaling pathway might be activated in dysplastic tissue but not SCC. These results indicate HPV type 6 is not capable of mediating a malignant transformation but that it can potentially increase cell proliferation. This increased cell proliferation in turn facilitated a significant genetic defect leading to malignant transformation so that p53 staining became positive.

It is noteworthy that the multi functional tumor suppressor gene, p53, was not expressed in dysplastic tissue but was strongly expressed after malignant transformation to SCC. It is well-established that the E5 gene product of HPV types 16 or 18 can inactivate p53 function leading to malignant transformation (Kim et al. 2010) and that *p53* is wild type but not expressed in cervical cancers, although some cervical cancers do express mutant p53 and not infected by HPV. Unlike cervical cancers, malignant transformation in this case might have been contributed to by a mutation in p53, leading in turn to enhanced cell proliferative stimulation by HPV type 6 over an extended period. This is distinct, however, from a direct oncogenic effect of HPV.

The relationship between p53 expression and malignant transformation is still unclear. Some previous studies reported progressively increased expression of p53 and suggested that this was a significant event in the progression to SCC (Stern et al. 2000; Lin et al. 2010). Although our observation supports the concept that p53 is related to malignant transformation, another study reported that p53 protein overexpression was present in both papillomas and carcinomas at variable levels in the epithelium without any consistent pattern (Go et al. 2003). Taken together, it seems likely that there is no unique mechanism responsible for the malignant transformation of RRP and that the chronic proliferative stimulation resulting from HPV infection leads to genomic instability. Thus, these cancers may be very difficult to histologically and clinically diagnose early in the course of transformation.

Malignant transformation of RRP is almost impossible to treat. Antiviral agents and interferon have shown some promise, but unfortunately very mild curative effects. Therefore, novel treatments are needed for RRP before malignant transformation occurs, both to improve survival and to achieve a worthwhile, less toxic palliation. In this case, as well as in others previously described, EGFR signaling was activated in RRP, and is likely to have driven the growth of the papilloma. Wu et al. showed that, in papilloma-derived epithelial cells, EGFR, Rac1, and COX-2 act in a linear pathway with EGFR upstream of Rac1, which in turn results in increased expression of COX-2 (Wu et al. 2007). Inhibition of this pathway using the selective COX-2 inhibitor celecoxib reduced papilloma cell proliferation and increased the rate of apoptosis. Furthermore, a proof-of principle study of three patients with severe RRP treated with celecoxib showed disease remission in all cases (Lucs et al. 2012).

## Conclusion

Although further investigation is necessary before the clinical application of molecular targeted therapies, our results can serve as a foundation for the development of novel therapeutic strategies including molecular targeted therapy of EGFR signaling for severe RRP.

## Consent

Written informed consent was obtained from the patient for publication of this report and any accompanying images.
